# Maternal obesity and depression reported at the first antenatal visit

**DOI:** 10.1007/s11845-021-02665-5

**Published:** 2021-06-15

**Authors:** Emma H. Tuthill, Ciara M. E. Reynolds, Aoife McKeating, Eimer G. O’Malley, Mairead M. Kennelly, Michael J. Turner

**Affiliations:** UCD Centre for Human Reproduction, Coombe Women and Infants University Hospital, Dublin 8, Ireland

**Keywords:** Maternal depression, Maternal obesity, Postnatal depression, Pregnancy

## Abstract

**Background:**

Maternal obesity and depression are common and both have been associated with adverse pregnancy outcomes.

**Aims:**

The aim of this observational study was to examine the relationship between maternal body mass index (BMI) category and self-reported depression at the first antenatal visit.

**Methods:**

Women who delivered a baby weighing ≥ 500 g over nine years 2009–2017 were included. Self-reported sociodemographic and clinical details were computerised at the first antenatal visit by a trained midwife, and maternal BMI was calculated after standardised measurement of weight and height.

**Results:**

Of 73,266 women, 12,304 (16.7%) had obesity, 1.6% *(n* = 1126) reported current depression and 7.5% *(n* = 3277) multiparas reported a history of postnatal depression. The prevalence of self-reported maternal depression was higher in women who had obesity, > 35 years old, were socially disadvantaged, smokers, had an unplanned pregnancy and used illicit drugs. After adjustment for confounding variables, obesity was associated with an increased odds ratio (aOR) for current depression in both nulliparas (aOR 1.7, 95% CI 1.3–2.3, *p < *0.001) and multiparas (aOR 1.8, 95% CI 1.5–2.1, *p < *0.001) and postnatal depression in multiparas (aOR 1.4, 95% CI 1.3–1.5, *p < *0.001). The prevalence of current depression was higher in women with moderate/severe obesity than in women with mild obesity (both *p < *0.001).

**Conclusions:**

We found that self-reported maternal depression in early pregnancy was independently associated with obesity. The prevalence of depression increased with the severity of obesity. Our findings highlight the need for implementation of strategies and provision of services for the prevention and treatment of both obesity and depression.

**Supplementary information:**

The online version contains supplementary material available at 10.1007/s11845-021-02665-5.

## Introduction

Maternal obesity and depression in pregnancy are associated with adverse pregnancy outcomes. Maternal obesity, based on a body mass index (BMI) > 29.9 kg/m^2^, has emerged as a serious challenge in contemporary obstetrics [1–3]. It is associated with an increase in maternal complications, such as gestational diabetes mellitus (GDM) and preeclampsia, and an increase in foetal complications including preterm birth, macrosomia and congenital anomalies. The increase in pregnancy complications is associated with an increase in obstetric interventions, such as caesarean section and induction of labour. Excessive adiposity also poses technical difficulties, for example, with epidural analgesia, phlebotomy, foetal ultrasound and caesarean section. The increase in complications and interventions increase the economic costs for the maternity services [4]. While there is considerable data available on national and international rates of adult obesity, including on women of childbearing age, there is a dearth of high quality measured data on maternal obesity rates. A recent study from our hospital reported that about one in five women presenting for antenatal care is in the obesity category and that the rate has increased over the last decade [5]. Similar trends have been reported in other high income countries [6].

Maternal depression in pregnancy is associated with emotional and behavioural problems in the offspring [7, 8], and some studies suggest that it may be associated with low birth weight and preterm birth [9]. In a 2005 North American study of women, 9.1% of pregnant women met criteria for depression [10].

While some studies have demonstrated an association between maternal depression and obesity, the associations are too limited to draw solid conclusions. A systematic review by Molyneaux et al. and Steinig et al. including 39 and 14 papers, respectively, found that women in the obese weight category are more likely to experience depression symptoms [11, 12]. Molyneaux et al. [11] found that, when high quality studies only were included, women with obesity had an OR 1.41 (95% CI 1.28–1.54) compared with normal weight controls; however, this meta-analysis did not adjust for confounders. There is also a wide variation in how both BMI and depression are measured in the literature. Many studies rely on self-reporting of BMI and the use of screening questionnaires for depression. The purpose of this observational study in a large European maternity hospital was to examine the association between self-reported maternal depression reported at the first antenatal visit and maternal obesity measured accurately at the same visit.

## Methods

The hospital is the one of the largest maternity units in Europe, with over 8000 births annually [13]. Women from all socioeconomic groups across the urban–rural divide, whether privately insured or not, are accepted for antenatal care. The relationship between self-reported maternal depression and obesity was examined using data collected routinely at the first antenatal visit. Data for all women who delivered a baby weighing ≥ 500 g between 1 January 2009 and 31 December 2017 were included.

At the first visit, a trained midwife computerised answers given to standardised questions for each woman as part of the medical records. These questions remained unchanged over the study period. The questionnaire collected information on clinical and socioeconomic demographics and information on lifestyle behaviours. Data included age, parity, civil status, country of birth, planned or unplanned pregnancy, occupation, smoking, alcohol and illicit drug use and self-reported diagnosis of depression, anxiety and antidepressant/anxiolytic use.

The prevalence of self-reported depression, anxiety and antidepressant/anxiolytic use were based on the answers women gave to the standardised questions. All women were asked of the following: “have you ever suffered from any emotional or psychological problems?” The most appropriate answer/s were then selected: ‘no’, ‘depression in past’, ‘suicide attempt’, ‘depression now’, ‘nervous breakdown’, ‘puerperal psychosis’, ‘psychiatric problem’, ‘bulimia nervosa’, ‘anorexia nervosa’, ‘panic attacks’, ‘postnatal depression’, ‘other’, and ‘do not know’. If the patient answered ‘no’, no further questions were asked. If the woman had an emotional/psychological problem, they were asked “what treatment did you receive?” Answers were recorded as one of the following options: ‘no treatment given’, ‘treated by GP’, ‘mental health services OPD’, and ‘mental health services inpatient’. As part of the medical history, women were asked “did you use any medicine this pregnancy (prescribed/over the counter/herbal)?”.

Body mass index (BMI) was calculated after measurement of maternal height and weight at the same first antenatal visit. Height was measured in centimetres to one decimal point using a wall-mounted meter stick (Seca 242), and maternal weight was measured in kilograms to the nearest decimal point in a standardised way using digital weighing scales (Seca M). The same scales and methods were used in all antenatal clinics. Maternal BMI was categorised according to the World Health Organisation (WHO) categorisation of BMI, i.e., underweight (BMI ≤ 18.5 kg/m^2^), normal (BMI 18.5 kg/m^2^–24.9 kg/m^2^), overweight (BMI 25.0 kg/m^2^–29.9 kg/m^2^) and obesity class I (mild) (BMI 30.0 kg/m^2^–34.9 kg/m^2^), class II (moderate) (BMI 35.0 kg/m^2^ – 39.9 kg/m^2^) and class III (severe) (BMI ≥ 40.0 kg/m^2^).

Data were concurrently computerized onto the electronic system of the hospital ‘Euroking K2’. Data were pseudonymised by the IT department of the hospital before release of data to the researcher. Data were coded on a Microsoft Excel^©^ spreadsheet and stored in a secure data repository. Continuous variables were collapsed into categorical variables including age (< 35 years, ≥ 35 years) and parity (nulliparous, multiparous). Country of birth was classified as Ireland, European Union (EU) 14 (women born in the 14 other countries in the EU before 2004), EU 13 (women born in the 13 Accession countries that joined the EU following enlargement in 2004), or elsewhere (women born outside the EU). The woman’s occupation was used to categorize socioeconomic groups as professional/managerial, other non-manual or skilled manual, semi-skilled or unskilled manual, and unemployed.

The distribution of continuous data was assessed for normality by assessing the kurtosis and skewness of the distribution and the associated Kolmogorov-Smirnoff statistics, and by a visual inspection of the distribution histogram and boxplot. Descriptive statistics were first used to describe the characteristics of the study cohort. Inferential chi-square tests for independence were then used to analyse differences in categorical variables between different population groups.

Binary logistic regression analyses were performed to assess the independent association of a number of putative predictor variables with maternal depression, anxiolytic and antidepressant use, and postnatal depression. The model included independent variables that were selected due to their significant association with the dependant variables on univariate analyses (*p < *0.05).

Results were reported as proportions and means. A p value of < 0.05 was considered statistically significant. The analyses were performed using Statistical Package for Social Sciences (SPSS) version 24.0.0 (Chicago, Illinois). The study was approved by the Hospital Research Ethics Committee (study number 4–2013).

## Results

Table 1 shows the characteristics of the study population and the prevalence of self-reported current depression across the maternal BMI categories. This has been further subdivided for nulliparous and multiparous women available in supplementary documents Supplementary Table 1a and Table 1b. Of the 73,266 women, 12,304 (16.7%) were in the obesity category.

**Table 1:** Characteristics of the study population stratified by maternal BMI categories

	n	Underweight *n* = 1931	Normal weight *n* = 37,833	Overweight *n* = 21,198	Obesity *n* = 12,304	Total *n* = 73,266
Anxiety (%)	2704	4.7	3.6	3.6	3.9	3.7
Depression (%)	1192	1.6	1.3	1.6	2.7	1.6
Postnatal depression (%^)a^	3277	7.5	6.4	7.9	9.4	7.5
Antidepressants/anxiolytics (%)	1533	2.3	1.7	2.2	3.1	2.1
Age (years; mean, SD)						
Married/civil partnership (%)	47,083	59.9	65.3	65.2	60.4	64.3
Irish-born (%)	51,371	65.6	69.1	71.2	73.4	70.3
Infertility treatment (%)	2649	3.5	3.5	3.8	3.6	3.6
Planned pregnancy (%)	48,667	59.3	68.3	66.9	61.4	66.5
Professional/managerial employment (%)	18,720	23.3	28.9	25.2	17.4	25.7
Unemployed (%)	5559	11.6	7.3	7.2	8.9	7.6
Smoked in pregnancy (%)	9209	17.6	11.9	12.3	14.2	12.6
Any alcohol use in pregnancy (%)	1114	1.2	1.5	1.8	1.2	1.5
Illicit drugs in pregnancy (%)	1158	2.7	1.7	1.5	1.3	1.6

^a^Of multiparous women

The prevalence of depression was 1.3% for women with a normal BMI compared with 2.7% in women with obesity (Table 1). The prevalence of postnatal depression in multiparous women of normal BMI was 6.4%, compared with 9.4% for women with a BMI in the obesity category. Trends in the prevalence of self-reported depression, postnatal depression and anti-depressant/anxiolytic use each year from 2009 to 2017 are shown in Table 2.

**Table 2:** Trends in self-reported mental health status at the first antenatal appointment by year

	2009	2010	2011	2012	2013	2014	2015	2016	2017	Total
Depression (%)	1.5	1.5	1.5	1.6	1.6	1.5	1.6	1.6	2.3	1.6
Postnatal depression (%)	4.1	4.9	4.0	4.9	4.1	4.7	4.6	4.5	4.6	4.5
Antidepressants/anxiolytics (%)	1.6	1.9	1.9	2.0	1.9	2.2	2.2	2.2	3.0	2.1

Our results demonstrated a higher prevalence of self-reported depression, postnatal depression and antidepressant/anxiolytic use in women with severe forms of obesity (class III) compared with women in a milder obesity subcategory (class I) supporting evidence of a dose–response relationship (*p < *0.05) (Table 3).

**Table 3:** Current mental health status analysed by obesity categories stratified by parity

Parity	Mental health diagnosis (prevalence %)	*n*	Total	Obesity class I	Obesity class II	Obesity class III	*p*-Value
Nulliparous	Depression (%)	89	2.3	2.0	2.6	3.3	< 0.001
	Antidepressants/anxiolytics (%)	93	2.4	1.8	3.1	4.5	< 0.001
Multiparous	Depression (%)	242	2.9	2.6	3.6	3.4	0.003
	Postnatal depression (%)	786	9.4	8.8	10.3	10.7	0.021
	Antidepressants/anxiolytics (%)	293	3.5	3.2	3.9	4.5	0.032

The relationship between antidepressant and anxiolytic use (Table 4) and postnatal depression (Table 5) and maternal characteristics and lifestyle factors are shown. Women who were overweight compared to normal weight had a higher likelihood of antidepressant and anxiolytic use (crude OR 1.3, 95% CI 1.2–1.5, *p < *0.001), and higher likelihood of postnatal depression (crude OR 1.4, 95% CI 1.1–1.4, *p < *0.001). Women with obesity also had a higher likelihood of antidepressant and anxiolytic use (crude OR 1.9, 95% CI 1.7–2.2, *p < *0.001) and a higher likelihood of having a history of postnatal depression compared to women of normal weight (crude OR 1.5, 95% CI 1.4–1.6, *p < *0.001). These likelihoods remained significant (p $\le$ 0.001) after adjusting for confounding variables.

**Table 4:** The unadjusted and adjusted relationship between antidepressant and anxiolytic use and maternal characteristics and lifestyle factors

	Unadjusted		Adjusted	
	OR (95% CI)	*p*-Value	aOR (95% CI)	*p*-Value
BMI category				
Underweight	1.4 (1.0–1.9)	0.047	1.1 (0.8–1.6)	0.408
Normal weight	Reference	Reference	Reference	Reference
Overweight	1.3 (1.2–1.5)	< 0.001	1.2 (1.1–1.4)	0.001
Obesity	1.9 (1.7–2.2)	< 0.001	1.6 (1.4–1.8)	< 0.001
Age				
< 35 years	Reference	Reference	Reference	Reference
≥ 35 years	1.3 (1.2–1.5)	< 0.001	1.6 (1.4–1.8)	< 0.001
Nationality				
Irish-born	Reference	Reference	Reference	Reference
UK	1.1 (0.8–1.5)	0.481	1.0 (0.8–1.3)	0.954
EU 14	0.6 (0.4–0.9)	0.019	0.6 (0.4–1.0)	0.052
EU 13	0.2 (0.2–0.3)	< 0.001	0.3 (0.2–0.3)	< 0.001
Other	0.2 (0.2–0.3)	< 0.001	0.3 (0.2–0.4)	< 0.001
Parity				
Nulliparas	0.6 (0.6–0.7)	< 0.001	0.8 (0.7–0.9)	< 0.001
Multiparas	Reference	Reference	Reference	Reference
Employment status				
Professional/managerial	Reference	Reference	Reference	Reference
Other non-manual/skilled manual	1.6 (1.3–1.8)	< 0.001	1.4 (1.2–1.7)	< 0.001
Semi-skilled/unskilled manual	1.3 (1.0–1.7)	0.034	1.4 (1.1–1.9)	0.010
Unemployed	3.2 (2.7–3.9)	< 0.001	2.3 (1.9–2.8)	< 0.001
Homemaker	2.6 (2.3–3.1)	< 0.001	2.2 (1.8–2.6)	< 0.001
Student	1.7 (1.2–2.4)	0.003	1.7 (1.2–2.5)	0.003
Pregnancy intention				
Planned	Reference	Reference	Reference	Reference
Unplanned	2.0 (1.8–2.2)	< 0.001	1.5 (1.3–1.7)	< 0.001
Infertility treatment	0.6 (0.4–0.9)	0.012	0.6 (0.4–0.9)	0.015
Smoking status				
Never	Reference	Reference	Reference	Reference
Ex-smoker	1.9 (1.7–2.2)	< 0.001	1.6 (1.4–1.8)	< 0.001
Currently smoking	3.9 (3.4–4.4)	< 0.001	2.0 (1.7–2.4)	< 0.001
Alcohol use				
None	Reference	Reference	Reference	Reference
Current	2.3 (1.7–3.0)	< 0.001	1.5 (1.1–1.9)	0.013
Illicit drug use				
None	Reference	Reference	Reference	Reference
Current	5.0 (4.1–6.2)	< 0.001	3.0 (2.4–3.7)	< 0.001

Overall reference group: no antidepressant or anxiolytic use: *n* = 71,724. Adjusted model is mutually adjusted for all variables in the table

**Table 5:** The unadjusted and adjusted relationship between postnatal depression and maternal characteristics and lifestyle factors in multiparas *(n* = 43,890)

	Unadjusted		Adjusted	
	OR (95% CI)	*p*-Value	aOR (95% CI)	*p*-Value
BMI category				
Underweight	1.2 (0.9 − 1.5)	0.193	1.0 (0.9 − 1.4)	0.467
Normal weight	Reference	Reference	Reference	Reference
Overweight	1.4 (1.1 − 1.4)	< 0.001	1.2 (1.1 − 1.3)	< 0.001
Obesity	1.5 (1.4 − 1.6)	< 0.001	1.4 (1.3 − 1.5)	< 0.001
Age				
< 35 years	Reference	Reference	Reference	Reference
≥ 35 years	1.1 (1.0 − 1.2)	0.004	1.0 (0.9 − 1.1)	0.797
Nationality				
Irish-born	Reference	Reference	Reference	Reference
UK	1.0 (0.8 − 1.2)	0.862	0.9 (0.7 − 1.1)	0.426
EU 14	0.9 (0.7 − 1.3)	0.618	1.0 (0.7 − 1.4)	0.928
EU 13	0.4 (0.3 − 0.4)	< 0.001	0.4 (0.3 − 0.4)	< 0.001
Other	0.4 (0.4 − 0.5)	< 0.001	0.4 (0.3 − 0.4)	< 0.001
Employment status				
Professional/managerial	Reference	Reference	Reference	Reference
Other non-manual/skilled manual	1.7 (1.5 − 1.9)	< 0.001	1.5 (1.3 − 1.7)	< 0.001
Semi-skilled/unskilled manual	1.9 (1.6 − 2.2)	< 0.001	2.0 (1.7 − 2.4)	< 0.001
Unemployed	1.9 (1.6 − 2.2)	< 0.001	1.5 (1.3 − 1.8)	< 0.001
Homemaker	2.2 (2.0 − 2.4)	< 0.001	2.0 (1.8 − 2.2)	< 0.001
Student	1.4 (1.1 − 1.9)	0.020	1.6 (1.2 − 2.2)	0.002
Pregnancy intention				
Planned	Reference	Reference	Reference	Reference
Unplanned	1.6 (1.5 − 1.7)	< 0.001	1.4 (1.3 − 1.5)	< 0.001
Infertility treatment	0.7 (0.5 − 0.9)	0.027	0.7 (0.5 − 0.9)	0.047
Smoking status				
Never	Reference	Reference	Reference	Reference
Ex-smoker	1.6 (1.4 − 1.7)	< 0.001	1.3 (1.2 − 1.4)	< 0.001
Currently smoking	2.3 (2.1 − 2.5)	< 0.001	1.4 (1.2 − 1.5)	< 0.001
Alcohol use				
None	Reference	Reference	Reference	Reference
Current	1.4 (1.1 − 1.8)	0.003	1.1 (0.9 − 1.4)	0.362
Illicit drug use				
None	Reference	Reference	Reference	Reference
Current	2.3 (1.7 − 2.9)	< 0.001	1.4 (1.0 − 1.8)	0.023

Overall reference group: Multiparas with no reported postnatal depression: *n* = 40,613. Adjusted model is mutually adjusted for all variables in the table

The likelihood of self-reported current depression was higher among nulliparous women with obesity compared with those of a normal BMI (crude OR 2.0, 95% CI 1.5–2.5, *p < *0.001). Current depression was also more likely in women who were unemployed, semi- or unskilled, homemakers or students compared with professionally employed women, women with unplanned compared to planned pregnancy (crude OR 2.4, 95% CI 1.9 – 2.9, *p < *0.001), in women who were current smokers compared to never smokers (crude OR 4.2, 95% CI 3.3–5.4, *p < *0.001), women currently using illicit drugs compared to women who are not (crude OR 5.9, 95% CI 4.3–8.0, *p < *0.001) and women currently consuming alcohol compared to women abstaining from alcohol (crude OR 2.9, 95% CI 1.7–5.0, *p < *0.001). These associations remained significant after controlling for BMI, age, country of birth, maternal occupation, pregnancy intention, smoking status, alcohol consumption and illicit drug use (Table 6). Similar results were found for multiparas, with a higher likelihood of current depression with the same maternal characteristics and lifestyle factors discussed above for nulliparous women (Table 7).

**Table 6:** The unadjusted and adjusted relationship between self-reported current depression *(n* = 381) and maternal characteristics and lifestyle factors in nulliparas *(n* = 29,376)

	Unadjusted		Adjusted	
	OR (95% CI)	*p*-Value	aOR (95% CI)	*p*-Value
BMI category				
Underweight	1.3 (0.8 − 2.2)	0.345	1.0 (0.6 − 1.7)	0.946
Normal weight	Reference	Reference	Reference	Reference
Overweight	0.9 (0.7 − 1.2)	0.516	0.9 (0.7 − 1.1)	0.307
Obesity	2.0 (1.5 − 2.5)	< 0.001	1.7 (1.3 − 2.3)	< 0.001
Age				
< 35 years	Reference	Reference	Reference	Reference
≥ 35 years	1.2 (0.9 − 1.6)	0.075	2.0 (1.5 − 2.6)	< 0.001
Nationality				
Irish-born	Reference	Reference	Reference	Reference
UK	1.5 (0.9 − 2.5)	0.133	1.4 (0.8 − 2.4)	0.190
EU 14	0.9 (0.5 − 1.8)	0.873	0.9 (0.4 − 1.9)	0.800
EU 13	0.3 (0.2 − 0.5)	< 0.001	0.4 (0.3 − 0.7)	< 0.001
Other	0.7 (0.5 − 0.9)	0.040	0.9 (0.6 − 1.3)	0.429
Employment status				
Professional/managerial	Reference	Reference	Reference	Reference
Other non-manual/skilled manual	0.9 (0.6 − 1.4)	0.566	1.6 (1.1 − 2.1)	0.008
Semi-skilled/unskilled manual	2.3 (1.7 − 3.1)	< 0.001	1.4 (0.8 − 2.4)	0.186
Unemployed	1.6 (1.2 − 2.2)	0.002	2.4 (1.6 − 3.6)	< 0.001
Homemaker	2.0 (1.3 − 3.0)	0.001	2.4 (1.6 − 3.6)	< 0.001
Student	0.6 (0.4 − 0.8)	< 0.001	2.4 (1.4 − 3.9)	0.001
Pregnancy intention				
Planned	Reference	Reference	Reference	Reference
Unplanned	2.4 (1.9 − 2.9)	< 0.001	1.6 (1.3 − 2.1)	< 0.001
Infertility treatment	0.7 (0.4 − 1.3)	0.249	0.5 (0.3 − 1.0)	0.058
Smoking status				
Never	Reference	Reference	Reference	Reference
Ex-smoker	1.6 (1.2 − 2.0)	< 0.001	1.3 (1.0 − 1.7)	0.024
Currently smoking	4.2 (3.3 − 5.4)	< 0.001	2.4 (1.8 − 3.2)	< 0.001
Alcohol use				
None	Reference	Reference	Reference	Reference
Current	2.9 (1.7 − 5.0)	< 0.001	2.0 (1.2 − 3.6)	0.012
Illicit drug use				
None	Reference	Reference	Reference	Reference
Current	5.9 (4.3 − 8.0)	< 0.001	3.4 (2.4 − 4.8)	< 0.001

Overall reference group: nulliparas with no reported current depression: *n* = 28,995. Adjusted model is mutually adjusted for all variables in the table

**Table 7:** The unadjusted and adjusted relationships between self-reported current depression *(n* = 811) and maternal characteristics and lifestyle factors in multiparas *(n* = 43,890)

	Unadjusted		Adjusted	
	OR (95% CI)	*p*-Value	aOR (95% CI)	*p*-Value
BMI category				
Underweight	1.2 (0.7 − 1.9)	0.507	.09 (0.6 − 1.5)	0.755
Normal weight	Reference	Reference	Reference	Reference
Overweight	1.3 (1.1 − 1.5)	0.003	1.2 (1.0 − 1.4)	0.024
Obesity	2.0 (1.7 − 2.4)	< 0.001	1.8 (1.5 − 2.1)	< 0.001
Age				
< 35 years	Reference	Reference	Reference	Reference
≥ 35 years	0.9 (0.8 − 1.1)	0.208	1.2 (1.1 − 1.4)	0.007
Nationality				
Irish-born	Reference	Reference	Reference	Reference
UK	1.2 (0.8 − 1.7)	0.372	1.1 (0.7 − 1.6)	0.704
EU 14	1.3 (0.8 − 2.2)	0.300	1.7 (0.9 − 2.8)	0.056
EU 13	0.4 (0.3 − 0.6)	< 0.001	0.4 (0.3 − 0.6)	< 0.001
Other	0.6 (0.4 − 0.7)	< 0.001	0.6 (0.5 − 0.8)	< 0.001
Employment status				
Professional/managerial	Reference	Reference	Reference	Reference
Other non-manual/skilled manual	2.3 (1.7 − 2.9)	< 0.001	1.9 (1.4 − 2.5)	< 0.001
Semi-skilled/unskilled manual	2.7 (1.8 − 3.9)	< 0.001	2.3 (1.6 − 3.3)	< 0.001
Unemployed	5.3 (3.9 − 7.2)	< 0.001	3.0 (2.2 − 4.2)	< 0.001
Homemaker	4.7 (3.6 − 6.1)	< 0.001	3.3 (2.5 − 4.3)	< 0.001
Student	4.2 (2.5 − 6.9)	< 0.001	3.6 (2.1 − 6.0)	< 0.001
Pregnancy intention				
Planned	Reference	Reference	Reference	Reference
Unplanned	2.5 (2.2 − 2.9)	< 0.001	1.7 (1.5 − 2.0)	< 0.001
Infertility treatment	0.7 (0.4 − 1.4)	0.318	0.8 (0.4 − 1.6)	0.597
Smoking status				
Never	Reference	Reference	Reference	Reference
Ex-smoker	1.6 (1.3 − 1.9)	< 0.001	1.4 (1.2 − 1.7)	0.001
Currently smoking	4.1 (3.5 − 4.9)	< 0.001	2.1 (1.7 − 2.6)	< 0.001
Alcohol use				
None	Reference	Reference	Reference	Reference
Current	2.6 (1.8 − 3.7)	< 0.001	1.7 (1.2 − 2.4)	0.006
Illicit drug use				
None	Reference	Reference	Reference	Reference
Current	8.8 (6.6 − 11.7)	< 0.001	4.0 (2.9 − 5.4)	< 0.001

Overall reference group: nulliparas with no reported current depression: *n* = 43,079. Adjusted model is mutually adjusted for all variables in the table

Women with a weight in the underweight category had a higher prevalence of anxiety, current depression, postnatal depression and antidepressant/anxiolytic use compared with women of normal weight (Table 1). Women in the underweight category had a higher likelihood of anxiolytic and antidepressant use than women in the normal weight category (crude OR 1.4, 95% CI 1.0–1.9, *p =* 0.047); however, this was not sustained following adjustment for confounding variables (aOR 1.1, 95% CI 0.8–1.6, *p =* 0.408). There was no difference in their likelihood of reporting current depression (crude OR 1.3, 95% CI 0.8–2.2, *p =* 0.345 for nulliparous women and crude OR 1.2, 95% CI 0.7–1.9, *p =* 0.507, for multiparous women) or postnatal depression in multiparous women (crude OR 1.2, 95% CI 0.9–1.5, *p =* 0.193).

## Discussion

This large observational study supports an association between maternal obesity and depression. After adjusting for confounding variables, women with obesity were almost twice as likely to self-report current depression. A dose–response relationship was also identified within the obesity subcategories. The likelihood of current depression, antidepressant/anxiolytic use and postnatal depression was higher in women with moderate/severe obesity compared with women who had mild obesity (*p < *0.05). In a previous study, women with obesity were shown to have significantly elevated odds of depression when compared with overweight women as the reference group, and the odds of antenatal depression increased for each unit increase in pre-pregnancy BMI [14]. Our study adds evidence to support this dose–response relationship between antenatal depression and the severity of maternal obesity.

The key strengths of this study included a large observational study, the timing of BMI categorisation with concurrent self-reporting of diagnosed mental health problems and important clinical and socio-demographic confounders were adjusted for.

The study included all the obstetric population over 9 years rather than a selected cohort of women. The computerisation of the obstetric records using standardised questions and barcoded answers meant that we could adjust the odds ratios for important clinical and sociodemographic confounders. In our study, adjusting for socio-demographic factors slightly reduced the strength of the association between obesity and antenatal depression but the association remained significant. Low socioeconomic status in particular has been associated with both maternal obesity [5, 15] and depression [16–18]. One of 39 studies in a systematic review examining antenatal depression [11] and obesity provided adjusted OR and that was for ethnicity [19]. A more recent systematic review found that the comparability of study results was limited due to different confounding factors, with 10 of 14 studies reporting adjusted associations [12]. Analysis of data from the Avon Longitudinal Study of Parents and Children (ALSPAC) which adjusted for age, ethnicity, marital status, occupation, highest educational level, parity, singleton or multiple pregnancy, stressful life events during pregnancy and social support during pregnancy, alcohol consumption, tobacco smoking, drug use, and physical activity found that women with obesity (based on self-reported BMI) had a higher aOR of 1.39 (95% CI 1.05–1.84) for antenatal depression (based on a score > 12 using Edinburgh Postnatal Depression Scale (EPDS) at both 18 and 32 weeks of gestation) compared with normal weight women [14].

BMI was calculated at the first antenatal visit based on standardised measurement of weight and height by a trained midwife. This is important because categorisation based on self-reporting underestimates maternal obesity [20] and leads to incorrect BMI categorisation in a fifth of cases [21]. Furthermore, discrepancies in the underestimation of BMI between self-reported and measured weight have been shown to increase at successively higher BMI categories [22]. The majority of previous studies examining the risk of depression in mothers with obesity have categorised obesity based on self-reporting [11, 12]. Of 52 studies included in two systematic reviews collectively examining the relationship between obesity and depression in pregnancy [11, 12], three studies measured BMI in the first trimester, one measured BMI at 15-week gestation, eight were extracted from records, two were unspecified, 35 were based on self-reporting of pre-pregnancy weight at various time points during the antenatal period and three were based on self-reporting of pre-pregnancy weight postpartum.

There has been wide variation in the timing of relationship between BMI categorisation and the diagnosis of depression in previous studies. Our study recorded BMI category and self-reported diagnosed mental health problems at the same first antenatal visit. This is important because screening tools suggest a large increase in depression with advancing gestation [23]. Postnatal questionnaires also raise questions about recall bias.

Our study was based on self-reporting of a diagnosis of depression by the woman herself. This method has both strengths and limitations. Many previous studies finding evidence of an association between depression and obesity have based the end point of maternal depression on a wide variety of screening tools for depression [11, 12]. High levels of depressive symptoms assessed by screening tools may not meet the diagnostic criteria for major depressive disorder and vice versa. In a study of 13,314 pregnant women from the Avon Longitudinal Study, the prevalence of antenatal depression, based on a score > 12 using Edinburgh Postnatal Depression Scale (EPDS), was the lowest among women who were normal weight (based on self-reported pre-pregnancy BMI) when they became pregnant (7.6%), intermediate among overweight women (8.5%) and the highest among women who had obesity when they became pregnant (10.7%) [14]. Of 52 studies included in two systematic reviews collectively examining the relationship between depression and obesity in pregnancy [11, 12], two used diagnostic criteria, three were extracted from records and 47 used screening tools as a measure of depression. Validity of these screening tools remains uncertain in the obstetric population. Screening tools have been associated with overestimating the prevalence of depression compared with diagnostic interviews. Therefore, the practice of reporting the percentage of patients with scores above cut-off thresholds in screening questionnaires for depression as disorder prevalence potentially misinforms users of epidemiological evidence [24].

The major limitation of this study is that data regarding mental health diagnosis was obtained from hospital records based on self-reporting, and it was not possible to verify the diagnosis from primary or other healthcare records. Women may have inaccurately reported their psychological problems, perhaps due to concern about stigma associated with psychological diagnosis. They may also have recently ceased medication for depression or anxiety pre-conceptually and therefore not reported it. Furthermore, other variables included in our analysis were subject to self-reporting bias including alcohol consumption, smoking and illicit drug use [25, 26].

Although there is an epidemiological link between maternal obesity and antenatal depression, the reason for the association remains to be elucidated. There is evidence that the association between obesity and depression is bidirectional with behavioural, physiological, cognitive and social mechanisms that may be responsible for the pathway [27]. Although cross-sectional evidence is informative, it does not provide detailed insight into the exact mechanisms linking depression and obesity. A longitudinal meta-analysis reviewing the association in non-pregnant adults found that persons with obesity had a 55% increased risk of developing depression over time, whereas depressed persons had a 58% increased risk of developing obesity [28]. A longitudinal study of maternal BMI in successive pregnancies found that, of the nulliparous women in the overweight category, 20.6% developed obesity by the second pregnancy and the development of obesity was independently associated with taking antidepressants or anxiolytics and postnatal depression [29].

The association between maternal obesity and antenatal depression is a concern because about one in five women presenting for antenatal care has obesity, and the evidence shows the prevalence in high income countries is increasing [5, 6]. The co-morbid impact of depression and obesity may lead to a particularly high risk group. This raises questions about screening all women, including those with obesity for depression. The evidence to support screening during pregnancy is, however, not as strong as for postnatal depression [30]. In a small European study of 98 pregnant women with obesity, 27.1% had a depressed mood. Women with a depressed mood were less likely to spend time on moderate to vigorous physical activity (*p =* 0.03) as measured objectively by an accelerometer from 15 weeks of gestation [31]. Behavioural change interventions for pregnant or postpartum women with obesity (e.g., improving diet, increasing physical activity) may need to be tailored for women with poor mental health.

## Conclusions

This study adds to a growing body of literature on the association between maternal obesity and depression. The identification of the increased prevalence and risk of antenatal and postpartum mental disorders among women with obesity has important implications for clinical care and future research. This awareness could lead to prevention, early detection, and cotreatment for women at risk. Future research focusing on eliciting the multifactorial aetiologies of the pathway between maternal depression and obesity and increasing the evidence base for successful behaviour-change interventions during pregnancy could inform the implementation of strategies and provision of services for the prevention and treatment of both obesity and depression. This has the potential to improve clinical outcomes for both the woman and her offspring.

## Supplementary information

Below is the link to the electronic supplementary material.

Supplementary file1 (DOCX 16 KB)

## Data Availability

The data that support the findings of this study are available but restrictions apply to the availability of these data, which were used with permission for the current study, and so are not publicly available. Data are however available from the authors upon reasonable request and with permission of the Coombe Women and Infants University Hospital.
