# Supplementary material for: Maternal obesity and depression reported at the first antenatal visit
Source: Ir J Med Sci. 2021 Jun 15;191(3):1241–50. doi: 10.1007/s11845-021-02665-5 (PMC9135864; doi:10.1007/s11845-021-02665-5)
Supplement: Supplementary file 1 — Supplementary file1 (DOCX 16 KB) [file 11845_2021_2665_MOESM1_ESM.docx]

Supplementary Table 1a. Nulliparas. Characteristics of the study population stratified by maternal BMI categories

|  | n | Underweight  *n*=928 | Normal weight  *n*=16,764 | Overweight  *n*=7,766 | Obesity  *n*=3,918 | Total  *n*=29,376 |
| --- | --- | --- | --- | --- | --- | --- |
| Anxiety (%) | 1257 | 5.3 | 4.1 | 4.0 | 5.4 | 4.3 |
| Depression (%) | 381 | 1.5 | 1.2 | 1.1 | 2.3 | 1.3 |
| Antidepressants/anxiolytics (%) | 456 | 2.0 | 1.3 | 1.6 | 2.4 | 1.6 |
| Age (years; mean, SD) | 29376 | 27.3 (6.2) | 29.2 (5.7) | 29.9 (5.8) | 29.8 (5.8) | 29.4 (5.8) |
| Married/Civil Partnership (%) | 16804 | 53.6 | 58.6 | 57.6 | 51.8 | 57.2 |
| Irish-born (%) | 20185 | 60.8 | 65.6 | 71.9 | 79.1 | 68.9 |
| Infertility treatment (%) | 1684 | 4.7 | 5.1 | 6.5 | 7.2 | 5.7 |
| Planned pregnancy (%) | 18746 | 57.1 | 64.5 | 64.6 | 61.1 | 63.9 |
| Professional/managerial employment (%) | 7887 | 20.6 | 28.8 | 27.6 | 20.1 | 27.1 |
| Unemployed (%) | 2392 | 13.7 | 8.1 | 7.6 | 8.7 | 8.2 |
| Smoked in pregnancy (%) | 3253 | 15.1 | 10.7 | 10.5 | 13.0 | 11.1 |
| Any alcohol use in pregnancy (%) | 391 | 0.9 | 1.3 | 1.7 | 1.0 | 1.3 |
| Illicit drugs in pregnancy (%) | 726 | 3.8 | 2.5 | 2.4 | 2.3 | 2.5 |

Supplementary Table 1b. Multiparas. Characteristics of the study population stratified by maternal BMI categories

|  | n | Underweight  *n*=1003 | Normal weight  *n*=21,069 | Overweight  *n*=13,432 | Obesity  *n*=8,386 | Total  *n*=43890 |
| --- | --- | --- | --- | --- | --- | --- |
| Depression (%) | 811 | 1.7 | 1.4 | 1.9 | 2.9 | 1.8 |
| Postnatal depression (%) | 3277 | 7.5 | 6.4 | 7.9 | 9.4 | 7.5 |
| Antidepressants/anxiolytics (%) | 1077 | 2.5 | 2.0 | 2.6 | 3.5 | 2.5 |
| Age (years; mean, SD) | 43890 | 31.4 (5.7) | 32.5 (5.0) | 32.7 (5.0) | 32.7 (5.0) | 32.6 (5.0) |
| Married/Civil Partnership (%) | 30279 | 65.8 | 70.7 | 69.6 | 64.5 | 69.0 |
| Irish-born (%) | 31186 | 70.1 | 71.8 | 70.8 | 70.7 | 71.3 |
| Infertility treatment (%) | 965 | 2.3 | 2.3 | 2.2 | 2.0 | 2.2 |
| Planned pregnancy (%) | 29921 | 61.3 | 71.2 | 68.2 | 61.5 | 68.2 |
| Professional/managerial employment (%) | 10833 | 25.7 | 28.9 | 23.9 | 16.2 | 24.8 |
| Unemployed (%) | 3167 | 9.6 | 6.7 | 6.9 | 8.9 | 7.3 |
| Smoked in pregnancy (%) | 5956 | 19.9 | 13.0 | 13.4 | 14.7 | 13.6 |
| Any alcohol use in pregnancy (%) | 723 | 1.6 | 1.7 | 1.8 | 1.3 | 1.6 |
| Illicit drugs in pregnancy (%) | 432 | 1.7 | 1.0 | 1.0 | 0.9 | 1.0 |
